# Bi-Fluoride of Ammonium

**Published:** 1909-08-15

**Authors:** Joseph Head


### BI-FLUORIDE OF AMMONIUM.

BY JOSEPH HEAD, D.D.S.

For a number of years I have been much interested in the erosion of the teeth, and tried a series of experiments concerning the dissolution of the enamel by acids and the speeds at which they would do it.

One evening I dropped a tooth covered with tartar into commercial hydrofluoric acid, desiring to see how much of the tooth would be left in the morning. When I returned my surprise was great to find that all the tartar had been dissolved, and the tooth untouched. I was not then able to explain it, and cannot now. Tartar is largely composed of carbonate and phosphate of calcium, and the tooth structure is largely carbonate and phosphate of calcium, with perhaps one or two per cent, of fluoride of calcium. Some may shake their heads and say that the fluoride of calcium is the reason, but that does not explain why the internal atoms and molecules of the tooth should resist the action of the hydrofluoric acid, and why the molecules of tartar will not.

You can take a diamond, which is pure carbon, heat it red-hot, and solder it with hard solder without hurting it. Do that to a piece of charcoal, and it will rapidly disappear, although both are pure carbon.

For one or two of my patients whom I knew very well, I used a little pure hydrofluoric acid. The first one nearly coughed her head off, and received a bad burn. Although it was a good thing for tartar out of the mouth, it did not seem to be good for the tissues in the mouth. I will not burden you with a report of my experiments but I finally came to a mixture of bi-fluoride of ammonium so bland that it could be poured on the skin, but which will etch glass and will dissolve tartar without injuring the tooth. I made this chemical discovery and the next thing was to work out its therapeutic value. For about a year and a half I tested this substance and by some good luck which does not happen often to me in my experimenting, all the incidental factors that ordinarily go wrong, have gone the right way, and as a matter of fact, the present solvent is more powerful when put in the mouth around the tooth, than it is in a glass outside the mouth.

I take a rubber, platinum-pointed syringe, and being careful to put cotton on each side of the gum to protect the mucus membrane, I inject the solvent to the bottom of the pockets, allowing it to stay there for about two minutes. There will be a slight itching and blanching of the soft part, and perhaps a little pain, although in some cases none. At the end of two minutes I let the patient rinse the mouth and send him away. The next time the tartar that was there will have become very soft and it can probably be scooped out. The second time all but the microscopic bits can be removed and the third time the gum will be stimulated and caused to re-adhere to the tooth. I have had wonderful success with it and those who have tried it and followed my directions have had similar success. I cannot help feeling that properly used this will be one of the great boons in dentistry.

If this solvent is allowed to dry in large quantities on the outside of the gum, it will cause a burn like carbolic acid, but if all excess is immediately wiped away there will be no harmful effects.

Teeth so loose that I have been able to move them back and forth have, by an application of this solvent twice a week, been tightened in position in a couple of months.

Yesterday I saw a patient with a left lower central incisor which had been weeping pus for the last six years, though he had had all manner of treatment. I said: "I think I can guarantee that in two weeks there will be no more pus." He said if I did that, I would be doing more than he believed possible. I made one application, and when I asked him if he had had any more pus he said he was not sure. I made another application and he said: "Doctor, I cannot get any pus at all now." I made the next application yesterday, and I had difficulty in getting the point of the syringe down in the pocket, but I got blood.

It was a quick case of recovery and the gum was starting to re-adhere to the tooth. I do not say that we will always get healing of the pockets with this solvent, but I can count the success by the hundreds. It is so valuable to me that I feel I am almost in a dream when I tell you about it. I do hope you will all get as good results with it as I have.

I read a paper on this subject at the National Association last summer, describing exactly how I made it. It is made as followss: Take the hydrofluoric acid and neutralize it with carbonate of ammonium. Filter it and evaporate the solution to half its bulk. Fill up to the original bulk with the acid solution and evaporate once more to half its bulk. If it is not properly done, it crystalizes, and there is free acid which is very irritating. I had difficulty in getting people to make it. Several firms tried it and some tried to improve it for me. I thought it was rather improbable that they in two or three days could improve what it had taken me years to work out.

I am making arrangements with Mr. Adamson, of Baker & Adamson, Easton, Pa., to make it. He tells me that he will reproduce the solution up to 1-100 of one per cent. of its acidity. That will be satisfactory, and I hope within a month or so all may get a supply of it. If anyone does not understand how to apply it, if he will come to Philadelphia, I shall be glad to show him how to do it—and this is a real invitation.

THE PRESIDENT: When Dr. Head brought this solution before the National Association last summer, it was criticised by some who thought they knew more about hydrofluoric acid than he did, but the solution was tested by a committee, and found, as stated. We cannot realize at once what this means to us, but if it be true, as Dr. Head states it, and I see no reason to doubt it—does it not seem as though we were at the beginning of the rational and exact attack upon what is to us the greatest danger of all—pyorrhea? All the various schemes for treating pyorrhea depend for success upon the great care with which the tartar is removed. This dissolves that which cannot be reached. It seems to me we owe Dr. Head an immense debt of gratitude for bringing us face to face with the solution of one of the most difficult problems we have in modern dentistry.

Dr. Merritt: As one of the committee appointed by the National Association in Boston to investigate the claims made by Dr. Head, I may say that every contention made by him was fully borne out by the investigations of that committee, so far as it was possible outside of the mouth. The solution had a solvent action upon the tartar on the roots of the teeth that was little less than miraculous, and yet had no effect on the tooth. When one realizes the difficulty of removing these particles by instruments, and that it is only when they are removed completely and the root left smooth and polished that we can expect anything like good results then only can we appreciate the value of the drug which Dr. Head has told us about to-night.

Dr. R. G. Hutchinson, Jr.: I am delighted to have been present to-night for I was unable to attend the meeting of the National Association last summer and had not heard of Dr. Head's discovery. It seems as though we were approaching the millennium when we can apply a chemical agent which will act on tartar and not injure the tooth. I take it that Dr. Head does not intend that it shall be used for the removal of all deposits—the bulky deposits around the necks of the teeth and where a considerable amount would have to be applied.

It appears to me to be an adjunct to instrumentation. We know that the flow of pus can be stopped and a restoration to health established quickly in accessible places by the use of instruments. Such an application as this would be useful in pockets inaccessible to instruments where we cannot reach the point of suppuration. There are some conditions of pyorrhea where we have removed the deposits and yet we cannot retain the tooth. Nothing would make it tolerable to the tissues, and it must be removed; Nature is exfoliating it.

It would be a mistake for the profession at large to think that in this they have a panacea that can be used indiscriminately—that even a child could use. We must nut overestimate its value, rush ahead, and then because we fail, condemn it. Let us be careful and apply it in cases where good judgment says it is called for, but we should not abandon the instruments. In the bifurcation of molar teeth, and places which we cannot reach with instruments, I should think this preparation would be most valuable.

Dr. William B. Dunning: I was also on the committee in Boston to examine this preparation, and I think it would be interesting to refer to certain particulars which Dr. Merritt did not mention. We made the experiment with hydrofluoric acid, and also with the ammonium bifluoride. In examining a freshly extracted tooth covered with hard tartar some six hours after immersion in Dr. Head's preparation, the tartar was removed and had fallen to the bottom of the pan. Some particles remained, but could be removed with the finger nail. The enamel was highly polished, the cementum was unchanged, but there was a slight change in the exposed dentine. This was after six hours, which, is, of course, a much longer test than could be made in the mouth. Dr. Head tells me the effect on dentine is a negligable factor in its use. He says it makes no material difference; but it should be mentioned, as this is a matter of scientific interest.

As I remember, the tartar was reduced to a soft, cheese-like mass. It was not dissipated, and the term solvent may not exactly describe the action of the compound; perhaps the word "disintegrant"—I am not sure that such a word exists—might more accurately indicate the effect of its application. The tartar is disintegrated and in a condition to be washed away.

I would like to ask Dr. Head whether the tartar in the pocket is to be reduced to this soft mass and removed with the instruments or syringed out, or whether the tartar, having been reduced to this soft mass, is later by some physiological process absorbed or otherwise removed.

Dr. Head: I am glad Dr. Hutchinson brought out the points he did. I did not wish to reiterate too much, but in my paper I said all the skill one had for the treatment of pyorrhea would come into play—that instrumentation was to be considered of prime importance. I agree with everything he said about taking off the tartar with instruments, but I do say that this solvent properly used where one cannot do anything else, will absolutely cause the tartar to disappear in time. For instance, a patient recently said to me concerning a weak molar: "Doctor, every time I bite on that tooth I get a disagreeable taste, and I feel it is not clean underneath." I made an application three days in succession, and she said the disagreeable taste had disappeared, and that the tooth was comfortable. Whether I hypnotized her or not, I do not know; but that is what she told me, and if we can have a material by which we can hypnotize our patients, we should use it.

I know of another case in which there was a constant flow of pus between the second molar and the wisdom tooth. With instruments I got a great deal of tartar out, but I felt there was tartar that could not be reached down near the end of the roots. The soft parts were so sore when the patient first came he thought he was getting an abscess. After three application of the solvent the soreness disappeared and the gum assumed a normal condition, I could still get pus, but that was about two months ago. He came to me a couple of times a week, and there is now no pus, and the tooth is comfortable. The last time I treated him I got blood from apparently the adhesion of the gum to the tooth. I had no X-ray and cannot tell whether the tartar is there or not, but if it is, the tooth is fooled, and does not act as though it was there.

In treating these pockets, I have sometimes injected it into the canal of a tooth, so that it came through the pocket on the outside. So long as it is confined to the inside of the pocket and under the mucus membrane there will be very little irritation. There may be some pain for ten or fifteen minutes, but the patient will invariably say on the next visit that the tooth seems better.

Dr. Dunning asked me to speak of that cheesy part of the tartar that was left when the solution had expended its force. I can only say where that can be reached with the scaler it will be better to remove it. It will come off very easily. I believe that this solution acts in two ways—it dissolves the tartar and stimulates the cells of the peridental membrane. I think it dissolves the microscopic particles of tartar that may be deposited in the peridental membrane, and in that way causes healing. I remember a case four months ago, where the tooth was so loose that I would have taken it out if it had not been for this solvent. The patient came in twice a week, and now the tooth has become firm enough to chew on, and I think it will become absolutely firm. The solvent stimulates the cells in the peridental membrane so that they will eat up the tartar just as the cells in certain kinds of stimulation will eat up the root. In one case it is a stimulation to destruction, and in the other a stimulation to restoration of health.

Properly injected twice a week to the tip of the roots and allowed to stay there for two minutes, and the mouth rinsed —cases that would be otherwise hopeless will nine times out of ten get well.

THE PRESIDENT: Does Dr. Head use the gold or the platinum syringe point?

DR. HEAD: It makes no difference. I generally use the platinum.

THE PRESIDENT: Is the solution used full strength or diluted?

DR. HEAD: Full strength. The addition of water destroyes the value.

THE PRESIDENT: How much is injected at a time?

Dr. Head: I fill the pocket full. If any excess gets on the gum, it must be wiped off.

Dr. Merritt: Does it deteriorate?

Dr. Head: No; properly made, it will improve. It will be less irritating. Some solutions I made six months ago are a little blander and still as effective.

Dr. K. C. Smith: What kind of a bottle should it be kept in?

Dr. Head: Gutta percha or wax. If the bottle is pure gutta percha, it is all right, but if not the wax bottle is better.

Dr. Tracy: What is the chemical name?

Dr. Head Bi-fluoride of ammonium.—*The Journal.*

